# Analysis of deviant behaviors and family functions in the population at risk of internet addiction among primary and secondary school students in Chengdu city, Sichuan province of China

**DOI:** 10.3389/fpubh.2024.1498466

**Published:** 2024-11-27

**Authors:** Xiujuan Zhang, Xi Huang, Qiong Chen, Yan Song, Xixi Jiang, Junxia Zhou, Xiufang Zhao, Li Zhao

**Affiliations:** ^1^Department of Neonatology Nursing, West China Second University Hospital, Sichuan University, Chengdu, China; ^2^Key Laboratory of Birth Defects and Related Diseases of Women and Children, Sichuan University, Ministry of Education, Chengdu, China; ^3^West China School of Nursing, Sichuan University, Chengdu, China; ^4^Department of Pediatric Hematology and Oncology, West China Second University Hospital, Sichuan University, Chengdu, China; ^5^Department of Nursing, West China Second University Hospital, Sichuan University, Chengdu, China; ^6^West China Research Center for Rural Health Development, Sichuan University, Chengdu, China; ^7^Department of Health Policy and Management, West China School of Public Health, West China Fourth Hospital, Sichuan University, Chengdu, China

**Keywords:** internet addiction, deviant behavior, family function, primary and secondary school students, a cross-sectional survey analysis

## Abstract

**Background:**

This study explores the correlation between internet addiction (IA) and deviant behaviors among primary and secondary school students. It analyzes the impact of family functional factors, such as family relationships, parent–child communication, and parental control, on IA. The findings aim to provide a scientific basis for educators and parents to develop targeted preventive and intervention measures.

**Methods:**

A cross-sectional survey was conducted using self-administered questionnaires among 8,816 students from five primary and secondary schools in Chengdu. The questionnaire included basic information about students and their families, Young’s Internet Addiction Scale, Shek’s Deviant Behavior Scale, and the Chinese Family Assessment Instrument (C-FAI). Chi-square tests and *t*-tests were used to compare differences between the at-risk group for internet addiction and the normal population. Statistically significant variables were extracted and included in the hierarchical regression model, with the level of significance set at *α* = 0.05.

**Results:**

The detection rate of IA risk among primary and secondary school students was 14.11%, and the detection rate of deviant behaviors in this group was 85.21%. Deviant behaviors such as deceit (*r* = 0.201), running away from home (*r* = 0.215), and damaging others’ property (*r* = 0.209) showed a weak correlation with the risk of internet addiction, and the differences were statistically significant (*p* < 0.05). The results of the hierarchical regression analysis showed that all five dimensions of family environment and delinquency scores positively predicted internet addiction. Specifically, the change in R^2^ was 8.7% for mutual concern among family members and 9.7% for student delinquency behavior. Together, family environment and delinquency behavior explained 22.1% of the variance.

**Conclusion:**

Primary and secondary school students at risk of IA are more likely to exhibit deviant behaviors, which show a weak correlation with IA. Gender and grade level significantly impact the risk of IA, indicating that male students and those in higher grades require more attention. Strengthening family interventions, especially in areas of mutual communication, family conflict resolution, and parental control, can help prevent IA among primary and secondary school students.

## 1 Introduction

Internet Addiction (IA), also known as pathological or problematic internet use, is identified as a behavioral addiction. It manifests through a series of extensive symptoms due to excessive internet usage. These symptoms include uncontrollable impulses to access the internet, a deep immersion in the digital realm, challenges in curbing internet use, and consequent underperformance in academic and work settings (1). Previous research has indicated (2–5) that IA can impair memory and attention span, diminish cognitive and decision-making capacities, and lead to emotional dysregulation, such as anxiety and depression, as well as serious physical health issues, including reduced respiratory function. Internet Gaming Disorder (IGD), as a specific manifestation of IA, was first introduced in the Fifth Edition of the Diagnostic and Statistical Manual of Mental Disorders (DSM-5) and defined as a potential mental health disorder. It has since been classified by the World Health Organization (WHO) in the 11th Revision of the International Classification of Diseases (ICD-11) as “Gaming Disorder,” a condition related to addictive behaviors (6).

Adolescents and school-aged children are at high risk for IA (7, 8). According to data released in December 2023 by the Youth Rights Department of the Central Committee of the Communist Youth League and the China Internet Network Information Center (CNNIC) (9), there are 193 million minors using the internet in China, with a penetration rate of 97.2%; the penetration rate among primary school students is 95.1%, and it exceeds 99% for other school ages. This trend is also prevalent globally. For example, among adolescents in Hong Kong, over 26% of secondary school students exhibit symptoms of Internet addiction (10). Similarly, in South Korea, more than 90% of teenagers frequently engage in online gaming, with a significant proportion meeting the diagnostic criteria for IA or IGD (11). Due to their still-developing cognitive abilities, primary and secondary school students often lack a proper understanding and control over deviant behaviors and internet addiction (IA). This makes them particularly vulnerable to impulsive tendencies influenced by IA, which can negatively impact their attitudes and personalities—effects that are especially pronounced when exposed to violent video games (12, 13). Research indicates a potential link between IA and deviant behaviors among adolescents, such as theft and fighting, with frequent instances of these behaviors being associated with an increased likelihood of school dropout, early-adult depression, and substance abuse (14). Thus, it is crucial to explore whether IA can effectively predict deviant behaviors among school-aged children.

The family plays a pivotal role in managing adolescents’ internet usage. Effective family interventions can help adolescents reduce screen time, but moderation is essential, as overly strict controls may actually increase the risk of Internet Addiction (IA) (15, 16). A longitudinal study in Korea found that improvements in family functioning, such as enhanced parent–child relationships, can significantly lower the risk of IA in adolescents (11). This study aims to examine the relationship between IA and deviant behaviors in primary and secondary school students and to assess the impact of changes in family functioning on IA. The findings will offer theoretical support for predicting deviant behaviors and developing family-based IA interventions. Additionally, the study will compare results with those from other countries to identify commonalities and unique aspects of IA among Chinese adolescents.

## 2 Methods

### 2.1 Participants

Chengdu, a major city in western China, consists of 12 districts, 3 counties, and 5 county-level cities. Covering a total area of 14,335 square kilometers (with urbanized areas accounting for approximately 6.6%), it has a resident population of about 16.6 million, 74.4% of whom live in urban areas. As the capital of Sichuan Province, Chengdu is home to 623 primary schools, 317 junior high schools, and 156 K-9 schools. The baseline data for this study were collected from December 23, 2019, to January 13, 2020, as part of the “Positive Youth Development Program,” supported by the Tian Jiabing Foundation under the Jockey Club’s Youth Enhancement Scheme. To ensure sample representativeness, Chengdu was first stratified into different regions based on its economic profile. One district was then randomly selected from each stratum, and within each selected district, a school was chosen at random from the available list of schools. Ultimately, five schools were selected for participation: one primary school, one junior high school, and three K-9 schools. Of these, one school was located in the city center, two in the southern suburbs, and two in the northern suburbs. To capture a comprehensive range of data, we invited all first- to ninth-grade students willing to participate, with no specific inclusion or exclusion criteria. A total of 8,968 children and adolescents aged 6 to 16 years were recruited from the selected schools through stratified sampling, with 8,968 questionnaires distributed and 8,816 valid responses collected, resulting in a response rate of 96.90%. Among the participants, there were 4,546 boys (51.56%) and 4,270 girls (48.44%). Findings revealed that internet addiction behaviors were generally more prevalent among male students than female students. This study aims to understand the current state of positive youth development and associated psychosocial and behavioral issues, as well as to assess the effectiveness of health education and promotion programs in schools in fostering youth development and addressing psychological and behavioral challenges. Further details regarding the study design and methods are available in other publications (17).

### 2.2 Study design

This study used a cross-sectional survey design, employing a self-administered questionnaire completed by students in their respective classrooms. Prior to the survey, the research team provided a detailed explanation of the study’s purpose to both students and their legal guardians, emphasizing data confidentiality and the voluntary nature of participation. Legal guardians were required to sign a written informed consent form. The study received approval from the Medical Ethics Committee of ** University (approval number: K2020025). The questionnaire was administered in a classroom setting, with two trained research assistants present to supervise and ensure that all students had ample time to complete the questionnaire independently. For younger students, the homeroom teacher read each question aloud and provided clarification when students had questions. After completion, research assistants collected the questionnaires and conducted a quick review to ensure completeness; students were asked to refill any missing responses when necessary. For more detailed information on the study design and methods, please refer to previous publications (17).

### 2.3 Measurement tools

#### 2.3.1 Young’s internet addiction test

This study used the Internet Addiction Test (IAT), developed by Young in 1998 (18). The IAT has been widely applied internationally and has undergone multiple translations and cultural adaptations to assess IA across diverse cultural contexts. The IAT consists of 20 items, scored on a 5-point Likert scale, where 1 represents “rarely” and 5 represents “always.” The total score ranges from 20 to 100. The scoring criteria are as follows: a score of 20–49 indicates normal internet usage, 50–79 suggests a tendency toward internet addiction, and 80–100 indicates severe internet use problems or a clear state of internet addiction. For this study, individuals scoring between 50 and 100 were classified as high-risk for IA and analyzed accordingly (19). The IAT is a self-report measure with good reliability and validity, boasting a Cronbach’s *α* coefficient of 0.91 and a test–retest reliability coefficient of 0.96.

#### 2.3.2 Deviant behavior scale

This study employed the Deviant Behavior Scale, originally developed by Shek and his team in 1999 to assess adolescent deviant behaviors in family, school, and societal settings (20). The scale includes 12 items that cover illegal behaviors as well as behaviors that violate family, school, and societal norms. It employs a 7-point rating scale, where 0 represents “never” and 6 represents “more than 10 times.” Students with no deviant behaviors were classified into the normal group, while those exhibiting any deviant behaviors were classified into the risk group. Higher scores indicate a higher frequency of engagement in deviant behaviors, thus reflecting greater severity. The scale has undergone multiple cultural adaptations and has been validated in various studies involving Asian adolescent populations (21–23). In this study, the Cronbach’s *α* coefficient of the scale was 0.835, indicating high internal consistency, and the KMO value was 0.933, indicating that the scale is suitable for factor analysis.

#### 2.3.3 Chinese family assessment instrument

The Chinese Family Assessment Instrument (C-FAI) was developed by Shek and colleagues in 2002 specifically to assess family functioning within the Chinese cultural context (21). The C-FAI consists of five subscales: Mutuality, Communication, Conflict and Harmony, Parental Concern, and Parental Control, with a total of 33 items. Scoring is based on a 5-point Likert scale, where 1 represents “very similar” and 5 represents “very dissimilar,” with higher scores indicating poorer family environmental function. In this study, the C-FAI demonstrated excellent internal consistency and reliability, with a Cronbach’s *α* coefficient of 0.936.

### 2.4 Statistical analysis

In this study, Epidata 3.1 software was used for double data entry and review to ensure data accuracy and consistency. Data analysis was conducted using SPSS 25.0 software. Descriptive statistics for categorical data were presented as frequencies and proportions, and differences between groups were compared using the chi-square test. For continuous data, were presented as (X̅ ± *S*) deviation, and independent sample *t*-tests were employed to compare means between two groups if they met the assumptions of normal distribution and homogeneity of variance. When normality was satisfied but variances were unequal, a corrected *t*-test was used.

To analyze the association between deviant behavior in the IA risk group and the normal group, Spearman’s rank correlation coefficient was used to assess the strength of the association between two variables, with results presented as *r* and *p* values. In addition, this study conducted a hierarchical regression analysis on IA scores, using age, gender, origin (urban/rural), and grade as control variables, and the five dimensions of family environment and delinquency scores as independent variables. The significance level for hypothesis testing was set at *α* =0.05.

The significance level for hypothesis testing was set at *α* = 0.05. To address the issue of alpha inflation caused by multiple testing, Bonferroni correction was applied to reduce the risk of Type I errors. All *p* values were evaluated after applying the multiple testing correction, with the significance level still set at α = 0.05.

## 3 Results

### 3.1 Survey results on internet addiction behavior

Among 8,816 primary and secondary school students, 1,244 (14.11%) were identified as being at risk of IA. Analysis shows that the IA risk among male students is generally significantly higher than that of female students (*p* < 0.05), and the detection rate of IA is higher among rural students compared to urban students (*p* < 0.05). Further stratified analysis revealed that the detection rate of IA was significantly higher among middle school students compared to primary school students. Specifically, there was no significant fluctuation in the IA detection rate among students from grades 1 to 5 (*p* > 0.05). However, beginning from grade 6, the IA detection rate showed a significant increasing trend year by year (χ^2^ = 202.081, *p* < 0.05) (Table 1).

**Table 1:** Characteristics and detection rates of internet addiction behaviors in the population.

Group		Normal group			Risk group			t/χ^2^	*p*	Detection rate (%)
		Male	Female	Total (*N*)	Male	Female	Total (*N*)			
Total (*N*)		7,572			1,244					14.11
Age (years)		10.69 ± 2.28			12.12 ± 2.29			−20.426	<0.001	
Gender	Male	3,832			714			19.712	<0.001	15.71
	Female	3,740			530					12.41
Origin	Urban	2,523	2,431	4,954	417	317	734	8.051	0.005	12.9
	Rural	1,295	1,323	2,618	297	213	510			16.3
Grade	1	203	184	387	17	8	25	7.270	0.122	6.07
	2	200	182	382	21	18	39			9.26
	3	518	512	1,030	60	26	82			7.37
	4	537	512	1,049	66	31	97			8.46
	5	569	537	1,106	53	21	74			6.27
	6	590	544	1,134	75	44	119	202.081	<0.001	9.5
	7	465	478	943	100	85	189			16.7
	8	383	424	807	146	141	287			26.23
	9	367	367	734	176	156	332			31.14

### 3.2 Detection rate of deviant behaviors in the high-risk group for IA

The data indicate that the overall detection rate of deviant behaviors is 66.73%. Within the IA risk group, the detection rate of deviant behaviors is 85.21%, compared to 63.68% in the normal group. The difference between these groups is statistically significant (*p* < 0.05) (Table 2).

**Table 2:** Detection rates of deviant behaviors in primary and secondary school students at high risk of internet addiction.

Group	Normal group	Risk group	Total
Number of people	7,572	1,244	8,816
Number of people with deviant behavior	4,822	1,060	5,882
Detection rate of deviant behavior (%)	63.68	85.21	66.73
χ^2^	222.990		
*P*	<0.001		

Among the 12 types of deviant behaviors, deceit (*r* = 0.201), running away from home (*r* = 0.215), and damaging others’ property (*r* = 0.209) show a weak correlation with the risk of IA, with these differences being statistically significant (*p* < 0.05) (Table 3).

**Table 3:** Occurrence of deviant behaviors in primary and secondary school students at high risk of internet addiction.

Group	Normal group	Risk group	Group difference		Correlation		Total
	Number detection rate (%)	Number detection rate (%)	χ^2^	*p*	*r*	*p*	Number detection rate (%)
Theft	508 (6.71%)	252 (20.26%)	248.966	<0.001	0.168	<0.001	760 (8.625%)
Deceit	2,888 (38.14%)	830 (66.72%)	357.857	<0.001	0.201	<0.001	3,718 (42.17%)
Truancy	157 (2.073%)	146 (11.74%)	300.603	<0.001	0.185	<0.001	303 (3.44%)
Running away from home	354 (4.68%)	253 (20.34%)	408.833	<0.001	0.215	<0.001	607 (6.88%)
Damaging others’ property	719 (9.50%)	363 (29.18%)	384.523	<0.001	0.209	<0.001	1,082 (12.27%)
Fighting	785 (10.37%)	346 (27.81%)	290.808	<0.001	0.182	<0.001	1,131 (12.83%)
Engaging in sexual relationships	401 (5.30%)	145 (11.66%)	74.394	<0.001	0.092	<0.001	546 (6.19%)
Group fighting	339 (4.48%)	184 (14.79%)	203.677	<0.001	0.152	<0.001	523 (5.93%)
Swearing	3,776 (49.87%)	948 (76.21%)	298.001	<0.001	0.184	<0.001	4,724 (53.58%)
Staying out without permission	274 (3.62%)	178 (14.31%)	251.022	<0.001	0.169	<0.001	452 (5.13%)
Terrorizing with violence	593 (7.83%)	267 (21.46%)	225.527	<0.001	0.160	<0.001	860 (9.76%)
Sneaking into rooms without permission	224 (2.96%)	146 (11.74%)	204.761	<0.001	0.152	<0.001	370 (4.20%)

### 3.3 Assessment of family function in populations at risk for IA

The data indicate that the average family function score for the IA risk group is 80.78 ± 25.42, which is significantly higher than the average score for the normal group (60.91 ± 22.84). The difference between the two groups is statistically significant (Z = −25.898, *p* < 0.05). The scores for the five dimensions: Family Interactions, Communication, Conflict and Harmony, Parental Care, and Parental Control also show statistically significant differences between the two groups (*p* < 0.05) (Table 4, higher scores indicate poorer family functioning).

**Table 4:** Evaluation of family environment function.

Internet addiction	Normal group (Mean ± SD)	Risk group (Mean ± SD)	*Z*	*p*
Total Score	60.91 ± 22.84	80.78 ± 25.42	−25.898	<0.001
Mutuality	10.08 ± 4.96	13.63 ± 5.86	−20.230	<0.001
Communication	9.21 ± 4.94	12.57 ± 5.68	−19.724	<0.001
Conflict and harmony	18.96 ± 7.92	25.04 ± 8.47	−23.680	<0.001
Parental concern	12.29 ± 6.31	16.84 ± 6.93	−21.727	<0.001
Parental control	6.47 ± 3.51	7.88 ± 3.35	−13.663	<0.001

Higher family functioning scores indicate poorer family functioning, including the total score as well as scores across five dimensions: Mutuality, communication, conflict and harmony, parental concern, and parental control.

### 3.4 Hierarchical multiple regression analysis of the impact of family environment on IA population

The study conducted a hierarchical regression analysis on IA scores, using age, gender, origin (urban/rural), and grade as control variables, while the five dimensions of family environment and delinquency scores were included as independent variables. The results indicated that among demographic variables, being male, grade level, and rural background positively predicted internet addiction. After controlling for demographic variables and other factors, all five dimensions of family environment and delinquency scores positively predicted internet addiction. Specifically, the change in R^2^ was 8.7% for mutual concern among family members and 9.7% for student delinquency behavior. Together, family environment and delinquency behavior explained 22.1% of the variance (Table 5).

**Table 5:** Hierarchical multiple regression analysis of family environment influencing internet addiction population (*N* = 8,816).

Predictor variables	Model 1		Model 2		Model 3		Model 4		Model 5		Model 6		Model 7	
	*β*	*t*	*β*	*t*	*β*	*t*	*β*	*t*	*β*	*t*	*β*	*t*	*β*	*t*
Constant		14.430***		10.279***		10.225***		8.308***		8.283***		7.179***		8.551***
Gender	−0.099	−9.885***	−0.093	−9.849***	−0.098	−10.355***	−0.093	−9.947***	−0.090	−9.719***	−0.086	−9.285***	−0.050	−5.778***
Age (years)	0.067	1.613	0.039	0.973	0.036	0.905	0.044	1.124	0.042	1.089	0.044	1.129	0.020	0.544
Grade	0.270	6.462***	0.303	7.645***	0.301	7.634***	0.289	7.413***	0.289	7.434***	0.293	7.564***	0.273	7.561***
Urban/Rural	0.039	3.912***	0.018	1.937	0.017	1.748	0.015	1.606	0.013	1.351	0.012	1.341	0.020	2.334*
Mutuality			0.296	31.237***	0.182	12.482***	0.086	5.436***	0.075	4.762***	0.060	3.770***	0.037	2.480*
Communication					0.150	10.311***	0.111	7.613***	0.102	6.973***	0.111	7.549***	0.072	5.257***
Conflict and harmony							0.188	14.821***	0.140	9.373***	0.126	8.379***	0.106	7.550***
Parental concern									0.083	6.009***	0.077	5.535***	0.058	4.499***
Parental control											0.076	7.667***	0.058	6.283***
Deviant behavior													0.331	36.207***
*R^2^*	0.125		0.212		0.221		0.240		0.243		0.248		0.346	
*Adjusted R^2^*	0.124		0.212		0.221		0.240		0.243		0.248		0.345	
*∆R^2^*	0.125		0.087		0.009		0.019		0.003		0.005		0.097	
*F*	313.857***		975.744***		106.316***		219.652***		36.104***		58.784***		1310.969***	

*** *p* < 0.001, ** *p* < 0.01, * *p* < 0.05. “Gender: ‘Male’ as the reference group; Origin: ‘Rural’ as the reference group”.

## 4 Discussion

### 4.1 Analysis of the current status of IA

The results of this study indicate that the detection rate of individuals at risk for IA is 14.11%. This finding is consistent with the results obtained by Sun MX and Li H (24, 25) using different questionnaires on similar populations, highlighting the significant concern regarding IA among primary and secondary school students in China. Currently, research on IA, both domestically and internationally, primarily focuses on adolescents aged 12 and above, as well as university students. There is relatively less research and intervention targeting IA in primary school students, despite adolescents being a high-risk group for IA (26–28). It is important not to overlook the dependency on the internet among younger primary and secondary school students, and even those in lower grades.

The study’s findings show that the incidence rate of IA is higher in secondary school students compared to primary school students. Although the detection rate of IA risk does not fluctuate significantly among primary school students in grades 1 to 5, the lack of clear normative data prevents us from accurately assessing the addiction risk levels in primary school students. Some studies have suggested (12, 29) that deviant behavior levels follow an inverted U-shaped trajectory with age, where the incidence of deviant behaviors increases from childhood to early adolescence and then decreases, regardless of different upbringing methods. This study found that the incidence rate of IA increases year by year after grade 6. Whether the trend of IA behavior aligns with the occurrence of deviant behaviors if the survey scope is extended to high school and university students requires further research for confirmation.

This study indicates that the detection rate of IA risk is significantly higher among male students compared to female students (15.71% vs. 12.41%). Previous research has indicated (30) that boys are more likely to engage in massively multiplayer online role-playing games (MMORPGs) and violent games, whereas girls’ online activities tend to focus more on casual games, social interactions, messaging, and online shopping. However, some domestic scholars have found (24) that there is no significant gender difference in the IA risk among primary and secondary school students. With the rapid advancement of computer network technology, the integration of intelligent network systems into daily life has become increasingly close. Particularly during the COVID-19 pandemic, when people were largely confined to their homes and schools shifted their educational activities online, this reliance on the internet was further intensified. During adolescence, girls, with their heightened self-esteem and growing self-awareness, often prefer interacting and communicating with their peers, hoping to find a sense of belonging within peer groups as a means to maintain their self-esteem. The virtual online world holds a strong appeal for adolescent girls who are in a rebellious phase and are particularly curious. Moreover, the convenience of smart services might make it easier for girls with weaker self-control to fall into IA, becoming immersed in the virtual world. However, these observations are based on behavioral tendencies and still require further research to confirm the underlying mechanisms. Thus, whether there are significant gender differences in IA among primary and secondary school students requires more in-depth research evidence for validation.

The results of this study indicate a significant difference in the detection rate of IA risk between urban and rural students (12.9% in urban areas, 16.3% in rural areas), consistent with the findings of Wang et al. (31). However, earlier studies on urban–rural differences in IA have yielded inconsistent results (32, 33). It is generally assumed that the higher network penetration and stability, along with the abundance of network resources and devices in urban areas, would result in a higher addiction risk. Recent studies, however, have shown (9, 31, 34) that the internet penetration rate among rural minors has reached 96.5% (compared to 97.5% for urban minors). The widespread use of smartphones and the absence of parents contribute to rural left-behind children developing compensatory behaviors and a “let it be” attitude, leading to their immersion in the internet. The lack of family education and unreasonable command-style communication further exacerbates this issue, causing rural left-behind children to exhibit rebellious behavior in response to the lack of familial affection.

### 4.2 IA and deviant behavior

Deviant behaviors can negatively impact students’ future development, family relationships, academic performance, and even societal stability, potentially leading to significant economic costs. This study found that the detection rate of deviant behaviors was 63.68% in the normal IA group, compared to 85.21% in the high-risk group. Although the exact mechanisms linking IA and deviant behaviors remain unclear, previous research has suggested that factors such as sensation-seeking, peer influence, and IA are positively correlated in pairs, with peer effects and deviant behaviors being considered significant contributors to IA (24, 29). Koot (35) propose that adolescents often view the internet as a “safe haven” in social interactions, especially when facing rejection or isolation in real life. IA may increase adolescents’ susceptibility to negative peer influence, thereby fostering deviant behaviors. Furthermore, emotional and behavioral issues may serve as bridges between IA and deviant behaviors. Ken and Guan (36) highlighted that internalizing problems (e.g., anxiety, depression), when interacting with family dynamics, could heighten psychological stress in adolescents, making them more inclined to seek escape in online environments, which can lead to IA and subsequent externalizing behaviors (e.g., aggression). Our findings showed a weak correlation between IA risk and certain deviant behaviors—such as deceit, running away from home, and property damage (*r* = 0.201, 0.215, and 0.209, respectively)—further suggesting that IA may be a predictive factor for certain deviant behaviors. This weak correlation may be attributed to differences in IA classification criteria and the complex psychological and environmental factors underlying deviant behaviors. Future research should further explore the role of family functioning, internalizing, and externalizing issues as mechanisms connecting IA and deviant behaviors, to deepen our understanding of the complex interactions between these behaviors.

### 4.3 Family function interventions for populations at risk of IA

The findings of this study indicate that individuals at high risk for IA score significantly lower in family functioning than those at normal risk, suggesting that poor family functioning may be a contributing factor to IA. Existing research highlights the importance of emotional support, communication quality, and moderate parental control in influencing adolescent behavior. Ken and Guan (36) noted that a lack of emotional support and family conflicts can intensify adolescents’ internalizing issues, such as anxiety and depression, which in turn may lead them to rely on the internet as a coping mechanism. Additionally, Jo et al. (11) found that the frequency and duration of internet use are closely linked to IA, as frequent internet use can serve as a means for adolescents to escape real-life challenges. Our study further reveals that mutual care among family members, open communication, harmonious conflict resolution, parental warmth, and appropriate parental control are significant factors influencing IA risk, with communication quality having a particularly notable impact. Although frequent communication can enhance parent–child relationships, if the content of these interactions is filled with conflict or negativity, it may inadvertently increase IA risk. This aligns with findings by Ken and Guan, who observed that negative family interactions could drive adolescents toward excessive internet use as an escape from reality (36). Furthermore, high levels of family conflict and an inhospitable family environment may prompt adolescents to use the internet as a way to vent emotional stress, thus exacerbating IA risk. Excessively strict parental control can also foster adolescent resistance, further increasing the likelihood of IA. Koot (35) found that in adolescents at risk for IA, moderate parental monitoring and emotional support serve as protective factors against excessive internet use.

In summary, this study highlights the critical role of family functioning in providing emotional support and behavioral management for adolescents at high risk for IA. Future interventions should focus on enhancing family emotional support and improving communication quality to reduce IA risk, rather than solely relying on restrictions on screen time. By detailing specific aspects of family functioning, this study provides a theoretical foundation for developing more targeted family-based interventions.

### 4.4 Practical implications and intervention recommendations

The findings of this study hold significant practical implications, particularly for the prevention and intervention of IA and related problematic behaviors among adolescents.

1. Enhancing the Quality of Family Communication: Studies indicate that improved family functioning, such as stronger parent–child relationships and effective communication, is associated with a reduced risk of IA (10). This study, however, found that while frequent family communication may help reduce IA risk, communication that is negative or conflict-laden can have the opposite effect. Therefore, interventions should focus on improving the quality of family communication and fostering constructive, supportive dialogues. Family education programs can equip parents with effective communication skills, help them build positive relationships with their adolescents, and guide them in managing their children’ s internet use without straining the parent–child relationship, thus reducing conflict-laden interactions.
2. Promoting Family Harmony and Reducing Conflict: This study found that frequent family conflicts and an overall disharmonious family environment are significant predictors of IA risk. Based on this finding, interventions can encourage families to foster a positive family atmosphere, utilizing family counseling or therapy to help minimize internal conflicts and create a harmonious environment for adolescent development.
3. Reasonable Parental Guidance and Monitoring: A longitudinal study in Hong Kong suggests that promoting positive youth development, such as cognitive and social skills, can effectively reduce IA behaviors (10). This study also found that overly strict parental control can increase IA risk. Therefore, interventions might include school or community-based programs to develop adolescents’ self-management and time-management skills. For example, offering courses on focus training, critical thinking, and time management could help adolescents control their internet use and reduce dependency. Additionally, parents could be trained in balanced monitoring and guidance techniques, such as setting reasonable internet usage rules while allowing a degree of autonomy. Parent workshops or community education programs can help parents provide adequate oversight without resorting to excessive control.
4. Targeted Support for High-Risk Groups: Since male students, rural students, and middle school students are at a higher risk of IA, interventions should specifically address these high-risk groups. Systematic mental health education and family support programs in rural areas or secondary schools could help these groups manage IA risk. Furthermore, a study in South Korea found that limiting adolescents’ online gaming time on weekdays (recommended at no more than 1.5 h per day and 4 days per week) can help reduce the risk of Internet Gaming Disorder (IGD) (11). Thus, targeted interventions might include setting daily or weekly internet usage limits at home or school and promoting healthy alternative activities, such as sports or outdoor pursuits, to redirect adolescents’ focus and reduce dependence on online entertainment.

In conclusion, this study suggests specific intervention measures, including enhancing the quality of family communication, reducing family conflict, implementing balanced parental guidance, and providing targeted support for high-risk groups. Additionally, promoting positive youth development through school and community activities, combined with managing internet use through structured time limits, can help reduce IA behaviors. These measures can not only assist adolescents in better managing their internet habits but also mitigate the psychological and behavioral issues arising from excessive internet use.

## 5 Conclusion

This study highlights the significant impact of family functioning on the risk of IA among adolescents, particularly in areas of emotional support, communication quality, and parental control. Our findings suggest that poor family functioning may increase the likelihood of IA in adolescents, and that high-risk IA groups exhibit a notably higher incidence of deviant behaviors. Improving the family environment, optimizing parent–child communication, and implementing moderate monitoring of internet use may help reduce the risk of IA, thereby supporting adolescents’ mental health and behavioral development. These findings offer practical recommendations for stakeholders in education and mental health. Educators can incorporate information on the influence of family functioning on IA into health education curricula, helping students and parents understand the importance of a supportive family environment. Mental health professionals can design family-based interventions for adolescents at high risk of IA, aiming to enhance communication patterns, reduce family conflict, and, where necessary, provide structured guidance on internet use. Additionally, policymakers can support community and school-based family education programs to equip parents with effective parenting and communication skills, thereby contributing to IA prevention in adolescents. Future research should further explore the specific mechanisms through which different aspects of family functioning affect IA and conduct longitudinal studies to examine the long-term association between family functioning and adolescent IA, offering a deeper understanding of the dynamic nature of these relationships.

## 6 Limitations and prospects

The study used self-reported questionnaires for data collection, which may introduce recall bias and social desirability bias. Participants may have been inclined to provide answers that align with social norms or expectations, potentially leading to underestimation or overestimation of actual behaviors. However, given the large-scale cross-sectional design of this study, self-reporting remains a cost-effective and practical approach and has been shown to be reasonably valid in assessing IA behaviors. Future research could consider incorporating multiple data collection methods, such as parental reports or observational methods, to improve data accuracy and reliability. Additionally, this study did not track the dynamic changes in IA behaviors and deviant behaviors over time, limiting our understanding of these behaviors’ development. Future studies could conduct longitudinal tracking and intervention research on individuals at risk of addiction, further analyzing the interaction and consistency between IA behaviors and deviant behaviors. These improvements would allow for a more comprehensive understanding of IA behaviors and their related factors over time.

Despite the increasing concern over IA behavior with the widespread use of the Internet, the positive aspects of the internet should not be overlooked. For instance, different types of online games offer varied human-computer interaction experiences, and cognition-based online games can enhance children’s attention, responsiveness, hand-eye coordination, and critical thinking skills (37). Experiential learning is often the most enduring and impactful. Hence, in assessing and intervening in IA behavior, it is crucial to understand the definition and diagnosis of IA behavior, particularly in family interventions where parents’ recognition and assessment of IA behavior are paramount. Moreover, integrating research on internet applications with students’ academic performance and cognitive development can guide students to use the internet correctly and responsibly.

## Data Availability

The original contributions presented in the study are included in the article/supplementary material, further inquiries can be directed to the corresponding authors.

## References

[1] Block JJ. Issues for DSM-V: internet addiction. Am J Psychiatry. (2008) 165:306–7. doi: 10.1176/appi.ajp.2007.07101556 18316427

[2] Näsi M Koivusilta L. Internet and everyday life: the perceived implications of internet use on memory and ability to concentrate. Cyberpsychol Behav Soc Netw. (2013) 16:88–93. doi: 10.1089/cyber.2012.0058, PMID: 23113691

[3] Tu XL Lin YX Yan JL Zhang GH. The moderating role of self-control in the relationship between impulsivity and risky decision-making among college students with pathological internet use. Chin J Behav Med Brain Sci. (2019) 28:930–4. doi: 10.3760/cma.j.issn.1674-6554.2019.10.013

[4] Zhang SH Yao X Zhang L Wu D Chen DM Zhang HB. The correlation between internet addiction and mental health among middle school students in Chengdu. Chin J Prev Control Chronic Dis. (2021) 29:37–40. doi: 10.16386/j.cjpccd.issn.1004-6194.2021.01.008

[5] Chen X Deng SL Chen G. Analysis of the correlation between physical health and internet addiction among college students. J Hebei Normal Univ. (2019) 43:546–52. doi: 10.13763/j.cnki.jhebnu.nse.2019.06.014

[6] Li GY Wang SM Yu MC Xu YS Ma HX. Comparison of mental, behavioral and neurodevelopmental disorders between the eleventh revision and the tenth revision of the international classification of diseases. Chin J Diagn. (2021) 9:217–20. doi: 10.3877/cma.j.issn.2095-655X.2021.04.001

[7] Tateno M Teo AR Ukai W Kanazawa J Katsuki R Kubo H. Internet addiction, smartphone addiction, and hikikomori trait in Japanese Young adult: social isolation and social network. Front Psych. (2019) 10:455. doi: 10.3389/fpsyt.2019.00455, PMID: 31354537 PMC6635695

[8] Park SK Kim JY Cho CB. Prevalence of internet addiction and correlations with family factors among south Korean adolescents. Adolescence. (2008) 43:895–909. PMID: 19149152

[9] Department of Youth Rights and Welfare of the Communist Youth League Central Committee, China Internet Network Information Center (CNNIC). (2023). The 5th National Research Report on internet usage among minors. Available at: https://qnzz.youth.cn/qckc/202312/P020231223672191910610.pdf (Accessed August 30, 2024).

[10] Yu L Shek DT. Internet addiction in Hong Kong adolescents: a three-year longitudinal study. J Pediatr Adolesc Gynecol. (2013) 26:S10–7. doi: 10.1016/j.jpag.2013.03.010 23683821

[11] Jo SJ Yim HW Jeong H Son HJ Lee HK. Prospective association between online game use and risk of internet gaming disorder in Korea: 12-month follow-up results from the iCURE study. Asia Pac J Public Health. (2022) 34:370–6. doi: 10.1177/10105395221084929, PMID: 35285284

[12] Munno D Saroldi M Bechon E Sterpone SCM Zullo G. Addictive behaviors and personality traits in adolescents. CNS Spectr. (2016) 21:207–13. doi: 10.1017/S1092852915000474 26268304

[13] Li CX. The impact of sensation seeking on aggressive behavior among high school students: The chain mediation effect of deviant peer affiliation and internet gaming addiction. Hebei: Hebei Normal University (2021).

[14] Cook EC Pflieger JC Connell AM Connell CM. Do specific transitional patterns of antisocial behavior during adolescence increase risk for problems in young adulthood? J Abnorm Child Psychol. (2015) 43:95–106. doi: 10.1007/s10802-014-9880-y, PMID: 24893667 PMC4256141

[15] Tang TC Ko CH Yen JY Lin HC Liu SC Huang CF. Suicide and its association with individual, family, peer, and school factors in an adolescent population in southern Taiwan. Suicide Life Threat Behav. (2009) 39:91–102. doi: 10.1521/suli.2009.39.1.91, PMID: 19298154

[16] Keijsers L Branje S Hawk ST Schwartz SJ Frijns T Koot HM. Forbidden friends as forbidden fruit: parental supervision of friendships, contact with deviant peers, and adolescent delinquency. Child Dev. (2012) 83:651–66. doi: 10.1111/j.1467-8624.2011.01701.x, PMID: 22181711

[17] Zhao L Shek DTL Zou K Lei Y Jia P. Cohort profile: Chengdu positive child development (CPCD) survey. Int J Epidemiol. (2021) 51:e95–e107. doi: 10.1093/ije/dyab237 35020880

[18] Young K Pistner M O'Mara J Buchanan J. Cyber disorders: the mental health concern for the new millennium. Cyberpsychol Behav. (1999) 2:475–9. doi: 10.1089/cpb.1999.2.475, PMID: 19178220

[19] Király O Nagygyörgy K Koronczai B Griffiths MD Demetrovics Z. Assessment of problematic internet use and online video gaming. In: Aboujaoude E Starcevic V, editors. Mental health in the digital age: Grave dangers, great promise. Oxford, UK: Oxford University Press (2015). 46–68.

[20] Shek DTL Zhu X. Paternal and maternal influence on delinquency among early adolescents in Hong Kong. Int J Environ Res Public Health. (2019) 16:1338. doi: 10.3390/ijerph16081338, PMID: 31013967 PMC6518268

[21] Shek DTL Ma CMS. The Chinese family assessment instrument (C-FAI) hierarchical confirmatory factor analyses and factorial invariance. Res Soc Work Pract. (2010) 20:112–23. doi: 10.1177/1049731509355145

[22] Shek DT Yu L. Prevention of adolescent problem behavior: longitudinal impact of the project P.A.T.H.S. In Hong Kong. ScientificWorldJournal. (2011) 11:546–67. doi: 10.1100/tsw.2011.33, PMID: 21403974 PMC5596514

[23] Sun RC Shek DT. Life satisfaction, positive youth development, and problem behaviour among Chinese adolescents in Hong Kong. Soc Indic Res. (2010) 95:455–74. doi: 10.1007/s11205-009-9531-9, PMID: 20062815 PMC2801834

[24] Sun MX. The relationship between parent-child communication and internet addiction among primary and secondary school students: The mediating effect of loneliness and the moderating effect of online self. Shandong: Liaocheng University (2021).

[25] Li H Ma YH. A study on the tendency of internet addiction and its influencing factors among primary and secondary school students in Daxing District. Beijing Capital Public Health. (2019) 13:40–2.

[26] Lee JY Kim SY Bae KY Kim JM Shin IS Yoon JS. Prevalence and risk factors for problematic internet use among rural adolescents in Korea. Asia Pac Psychiatry. (2018) 10:e12310. doi: 10.1111/appy.12310, PMID: 29356370

[27] Balhara YPS Mahapatra A Sharma P Bhargava R. Problematic internet use among students in South-East Asia: current state of evidence. Indian J Public Health. (2018) 62:197–210. doi: 10.4103/ijph.IJPH_288_17, PMID: 30232969

[28] Schielein MC Tizek L Schuster B Ziehfreund S Liebram C Eyerich K. Always online? Internet addiction and social impairment in psoriasis across Germany. J Clin Med. (2020) 9:1818. doi: 10.3390/jcm9061818, PMID: 32545234 PMC7355796

[29] Ning K Zhu ZY Zhu Z. The peer effect and family moderating effect on adolescent internet addiction. World Econ Herald. (2021) 5:67–85.

[30] Bickham DS. Current research and viewpoints on internet addiction in adolescents. Curr Pediatr Rep. (2021) 9:1–10. doi: 10.1007/s40124-020-00236-3, PMID: 33457108 PMC7796811

[31] Wang HW Zhou XL Shen H Li R An QY Xu PL. A comparison of internet addiction between urban and rural students across three educational stages in Liaoning Province in 2017. Chin J Public Health. (2018) 34:1148–50.

[32] Gao YB Peng WB Chen WW. A survey on internet addiction among primary and secondary school students in Zhejiang Province. Chin J Ment Health. (2008) 22:627–7.

[33] Wang YL Wang JP Fu DD. An epidemiological survey on internet addiction among primary and secondary school student internet users. Chin J Ment Health. (2008) 22:678–82.

[34] Wang L. Case work intervention on internet addiction among left-behind children in rural areas under the psychosocial therapy model. Shandong: Shandong University (2023).

[35] Koot HM. Pathological internet use, aggression, and cyberbullying in children and adolescents with attention deficit hyperactivity disorder – editorial comment. Alpha Psychiatry. (2022) 23:74–5. doi: 10.5152/alphapsychiatry.2022.2210222, PMID: 36426293 PMC9597056

[36] Ken CS Guan NC. Commentary: internalizing problems in childhood and adolescence: the role of the family. Alpha Psychiatry. (2023) 24:93–4. doi: 10.5152/alphapsychiatry.2023.080523, PMID: 37440898 PMC10334622

[37] Granic I Lobel A Engels RC. The benefits of playing video games. Am Psychol. (2014) 69:66–78. doi: 10.1037/a0034857 24295515

